# A Memorial Address on the Late Dr. Henry Campbell Doughty

**Published:** 1899-05

**Authors:** Joseph Eve Allen

**Affiliations:** Augusta, Ga.


### A MEMORIAL ADDRESS ON THE LATE DR. HENRY CAMPBELL DOUGHTY.*

BY JOSEPH EVE ALLEN, M.D.,
AUGUSTA, GA.

On April 1, 1899, the Students Memorial Society presented to the Faculty of the Medical Department of the University of Georgia, a portrait of the late Dr. Henry C. Doughty who was for several years Demonstrator of Anatomy in the institution.

Prof. Joseph Eve Allen received the picture and delivered the following address in behalf of the medical faculty:

"*Mr. Speaker:*—In behalf of the Faculty I accept, with thanks, the portrait of Dr. Doughty you have just presented, and I assure you that we all commend and are in full accord with your association in its laudable object to perpetuate the memory of those distinguished alumni of this college who have finished their labors and left behind records that are worthy of emulation.

" Right and proper it is that you should place in these halls a memorial to Henry Doughty, for it is his due, and in him the student never had a warmer, closer, truer friend. A teacher of marked ability, so recognized, and held in high esteem by his associates; still he was so young, so lately from your ranks, that keenly he felt all your needs and difficulties, and shared in all your troubles and joys. He also was my friend; to me he unmasked his soul and showed the bottom of his deepest thoughts, and as I look upon the familiar features there so faithfully portrayed, the past comes back and many things are present to my mind and press for utterance. I could tell of lofty aspirations, glorious day-dreams, struggles, hopes, and other matters such as go to make the woof and warp of genius; but these have all come to naught and are too sacred now to be disclosed. I prefer to point you to the lesson of his life—a lesson bright and clear and fraught with good to all who heed it; a lesson that teaches unflagging enthusiasm in the cause of science, unswerving devotion to the discharge of duty, philo-

*Delivered before the Faculty and Students of the Medical Department of the University of Georgia.

sophic patience in enduring suffering, and heroic courage in the face of death.

"He fell asleep ere manhood morn had brightened into noon, and yet his life was full of deeds that make men great. Not deeds that gain the praise and plaudits of the world, but deeds of kindness, sacrifice and service—deeds that endear him to the hearts of friends and raise him far above the plane of common men. Oft throughout the dark and stillness of the night he nursed the dying pauper back to health again—oft has his skill and sound advice turned to success the blundering work of others—oft has his helping hand aided the halting on life's rugged road, and many a weary sufferer's lot has oft been gladdened by his smile and word of kindly cheer.

"Henry Doughty—we all knew and loved him. Only a short time since he went in and out among us; but yesterday we stood with bowed heads and sorrowing hearts around his open grave. Sympathetic in nature, noble in every impulse, upright in his dealings, in speech bright, earnest and entertaining, a most companionable man, a born teacher, an accomplished physician, a true and generous friend, proud indeed am I to have been selected on this occasion to bear public witness to his work and worth.

"Our dean in his eloquent eulogy pronounced before you on the day of Dr. Doughty's interment, spoke truly when he said his death was nothing short of a calamity to this institution. For it he unceasingly planned and wrought; and to advance its interests was always one of the chief aims and objects of his life. The fidelity, ability, and zeal which he displayed in his department did much to elevate the curriculum and raise our school to its present high status among the medical colleges of the country.

"Dr. Doughty's death was a great loss to our profession, for he was ever just and loyal to his brethren. Liberal and broad-minded, he was superior to the petty bickerings, jealousies and strife that mar and embitter many a doctor's days. It may be truthfully said of him that there are few physicians in this community who did not feel a sense of personal loss at his decease, and owe him a debt of gratitude for services unselfishly performed.

"Henry Doughty came rightly by his talents. His was indeed a heritage of genius. Prof. Henry F. Campbell, his grandfather, under whose tutelage he was reared, and whose example was to him a continual stimulus, was one of the most eminent medical men the South has ever produced. An alumnus of this college and for years a member of this faculty, his many important scientific discoveries gave him a world-wide reputation, gained for him the highest honors of the profession and a name that will long endure in the annals of American medicine. This was the influence that induced Henry Doughty to take up the study of the healing art and pursue it with such indefatigable energy and zeal. He followed science for her own sake and not for any mercenary or other unworthy motive. He loved his work, and what to many is weariness and drudgery was to him a source of never-ending pleasure. His was a soul in full accord with all the harmonies of nature, and he never tired of seeking into her most recondite mysteries. The same enthusiasm that prompts the astronomer nightly to explore the celestial vault, searching for new worlds, or the biologist to pry into the depths of the dewdrop after its tiny tenants, caused him to study with probe and knife the wondrous mechanism of the human body. Animated by the ardor of a Versalius, he spent days and nights in the dissecting-room and the dead-house. For him these ghastly precincts had no horrors. A true scientist has no sentiment, and hence the dead outstretched there in him awakened no emotion, and presented nothing loathsome or repulsive. They were simply objects of intensely scientific interest from which might be learned much to benefit the living. The thorough anatomist soon became the skilful surgeon, and thus did his labors richly bring forth fruit.

"In the operating-room Henry Doughty was cool, calm, and imperturbable—some called him callous, but this was not the fact, for in sensibility he was as kind and tender as a woman. A perfect master of his art, the cold demeanor was a qualification essential to the work, for the surgeon must let nothing obscure the keen, clear glance of his science. He is unfitted for his mission if he allows aught to bias his judgment or unerve his hand. To him all who come, be they the young or the old, the beautiful or the decrepit, the innocent or the guilty, make but one appeal—the cry of human suffering seeking human aid.

"The world counts him a hero who falls amid the carnage and the smoke of battle, or yields up his life in the performance of some great exploit that dazzles by its daring. There are, however, other heroes, who die unhonored and unsung for the want of such dramatic setting to their taking off. The man who quietly and unnoticed does his duty, and in the service of his fellows fearlessly faces danger and meets his death, is just as great a hero as he who in dying gains the people's empty praises. Viewed in this light Henry Doughty was a hero. He contracted the dread disease that brought him to his end in attending on the sufferings of the poor. In the hovel and in the hospital ward the noxious seeds were sown, and his young life was worn out in ministrations that were unrewarded save by the commendations of his own conscience and the prayers and blessings of the wretched and the miserable. So full of promise and so soon cut off, he fell in the discharge of duty, a martyr to science and humanity, and though he may not be numbered among the great ones of the earth, still to him must be accorded the high honor of being written one who loved his fellowmen, for truly it is said, 'Greater love hath no man than this, that a man lay down his life for his friends.'

"While life was full of light and hope and love, and every portent promised happiness and joy, suddenly were all his aspirations and his efforts ended, and failure and bitter disappointment came to all his cherished hopes. The story of his days can be briefly written and soon told. In them, as people regard it, little was accomplished; but a man should be judged by his impulses and ambitions, and not by his actions, for actions are the result of circumstance, and accident often interferes with the execution of the best design. The impulse mirrors forth the man, and we should honor him for what he might have been, not for what he is or was. Therefore let charity cover up the faults and shortcomings of our friend, whatever they may have been, and let us join in the regret that a career so pregnant with beneficent possibilities should have come to such an untimely close, and all its good be lost forever to mankind.

"This life is a narrow path between great mountain heights, massive barriers that shut out all view beyond. We know not from whence this pathway comes or where it leads. Its beginning and its ending is wrapped in clouds of marvel, myth and mystery—clouds into whose shadow science sheds no ray and whose darkness is illumined only by the deceptive *ignis fatuus* of faith and hope. The end to every life, whether it comes in the sunniest hour of youth or in the season of the sear and yellow leaf, finds man's work unfinished and much yet before him to be done. The Psalmist says of human existence that even at fourscore years, 'It is soon cut off.' If failure marks the end of each and all, the pessimist may ask, Is life worth living; of what use is life? To this the scientist replies, Life is worth living. This is the end and object of our being. Each life is an important part of nature's plan, and every individual exerts an influence, little though it be, in the development and elevation of humanity. On this earth, 'nothing walks with aimless feet.' Each has his place and purpose in the evolution of the race. The mightiest force that affects man's upward progress is

* * * "'the choir invisible
Of those immortal dead who live again
In minds made better by their presence; live
In pulses stirred to generosity,
In deeds of daring rectitude, in scorn
For miserable aims that end with self,
In thoughts sublime that pierce the night like stars,
And by their mild persistence urge man's search
To vaster issues.'
"'This is life to come
Which martyred men have made more glorious
For us who strive to follow.'

"We will place this portrait of our brilliant and lamented friend on the walls of his alma mater that he loved so well, there with other of our illustrious dead to be forever an inspiration and an ideal to all who teach and all who learn within these portals."

